# Effects of financial incentives on motivating physical activity among older adults: results from a discrete choice experiment

**DOI:** 10.1186/1471-2458-14-141

**Published:** 2014-02-10

**Authors:** Muhammad Assad Farooqui, Yock-Theng Tan, Marcel Bilger, Eric A Finkelstein

**Affiliations:** 1 Health Services and Systems Research, Duke-NUS Graduate Medical School, 8 College Road, Singapore 169857, Singapore; 2 Global Health Institute, Duke University, 310 Trent Drive, Durham NC27710, USA

**Keywords:** Older adults, Physical activity, Incentives, Discrete choice, Conjoint

## Abstract

**Background:**

There is extensive evidence that regular physical activity confers numerous health benefits. Despite this, high rates of physical inactivity prevail among older adults. This study aimed to ascertain if incentives could be effective in motivating physical activity through improving uptake of walking programs, either with or without an enrolment fee to cover corresponding costs.

**Methods:**

A discrete-choice conjoint survey was fielded to a national sample of older adults in Singapore. Each respondent was given ten pairs of hypothetical walking programs and asked to choose the option they preferred. Each option varied along several dimensions, including the level and type (cash, voucher, or health savings credit) of incentive and an enrolment fee. For each option, they were asked how likely they would be to join their preferred program. A random utility model (RUM) was used to analyze the responses.

**Results:**

Results suggest that a free 6-month program with a $500 cash incentive would generate enrolment rates of 58.5%; charging $50 to enroll lowers this to 55.7%. In terms of incentive type, cash payments were the most preferred incentive but not significantly different from supermarket vouchers. Both were preferred to health savings credits and sporting goods vouchers. Concerns of adverse selection were minimal because those who were inactive represented at least 72% of new participants for any offered program(s) and were the majority.

**Conclusions:**

Study results demonstrate the potential for even modest incentives to increase program uptake among inactive older adults. Moreover, although cash was the most preferred option, supermarket vouchers, which could potentially be purchased at a discount, were a close alternative. Results also suggest that an enrolment fee is a viable option to offset the costs of incentives as it has only minimal impact on participation.

## Background

There is extensive evidence that physical activity exerts a strong protective effect on the health of older adults. This includes reducing the risk and progression of non-communicable diseases and other disabling conditions [1-4], improving cardiorespiratory and muscle fitness [5], health-related quality of life [6,7], psychological well-being [1] and longevity. It also has the potential to contain rising medical costs [8,9]. Despite these benefits, current data reveal low levels of physical activity levels among older adults [10,11] in nearly every developed country.

Walking is an accessible form of physical activity with proven health benefits [12] that can be conducted across a variety of settings without specialized equipment. It is also the most common form of physical activity among older adults who engage in regular activity [13]. It is for such reasons that walking programs have been promoted as a means to increase activity levels among sedentary older adults. Yet, in many communities, participation in structured programs targeting older adults remains low [14,15].

By increasing the benefits of participation, economic theory suggests that the use of incentives could increase participation rates in structured walking programs. Incentives have been shown to be effective in positively shaping various health behaviors and health outcomes [16-24] although there is limited data on its suitability and efficacy among older adults [25]. Moreover, even if effective, incentives come with a cost. One way to offset the cost is to include an enrolment fee. It is possible that the fee may deter some potential participants, but the net effect of incentives and an enrolment fee in affecting uptake by older adults in structured programs remains an empirical question. Therefore, the goal of this study was to conduct a conjoint analysis survey aimed at identifying the extent to which stated uptake of walking programs could be increased via the use of incentives, either with or without a corresponding enrolment fee.

In this stated preference conjoint study we estimate the net effect of several types/magnitudes of incentives and enrolment fees for candidate walking programs among a sample of older, multi-ethnic adults in Singapore. We hypothesize that incentives will increase the stated likelihood of participation in the programs regardless of the presence of an enrolment fee. Conjoint analysis is well suited for this task because it allows for generating predictions of program effects in a low-cost and efficient manner for several candidate programs, the most promising of which can then be tested in a randomized controlled trial to quantify the effects on both uptake and outcomes.

## Methods

### Participants and setting

The conjoint survey was administered in 2011 by a local survey firm to a national sample of 1,000 Singaporeans aged 50 and over. Trained interviewers took written consent and administered the paper and pencil survey in respondents’ homes. The interviews were conducted in four languages: English, Mandarin, Malay and Tamil.

### Survey structure

The survey contained questions concerning socio-demographic characteristics and health status. Data on past and present physical activity levels were also collected as were attitudes towards walking programs. The core of the survey was the discrete choice conjoint experiment. Each respondent was given ten pairs of hypothetical programs and asked to choose the option they preferred. They were then asked whether or not they would join their preferred program if given the option. If they responded “*Very likely”* or “*Somewhat likely*”, they were assumed to join their preferred program. Prior to fielding the conjoint questions, participants were introduced to the various program features and were told that the programs lasted for 6 months, were to be conducted in a group setting, and that each session comprised of 45 minutes of walking and 15 minutes of stretching, and that a non-refundable enrolment fee was to be paid in advance but that if they met program goals a financial incentive would be provided at the conclusion of the program. Other aspects of the programs varied along six dimensions (six attributes) as shown in Table 1.

**Table 1:** Attributes and levels

**Attributes**					
**Average number of sessions required per week**	**Cost to travel to sessions**	**Time to travel to sessions**	**Incentive payment at 6 months for meeting session goals**	**Type of incentive**	**Enrollment fee**
1 session	Free	15 minutes	$150	Cash payment	Free
2 sessions	$2	25 minutes	$200	Medisave account credit	$20
3 sessions	$5	30 minutes	$300	Supermarket voucher	$50
-	**-**	45 minutes	$450	Sporting goods voucher	-

All attributes and their levels were finalized through a series of focus groups and cognitive interviews. Travel time and related costs were included as travel considerations were likely to affect program participation. There were 4 levels for travel time ranging from 15 minutes to 45 minutes and 3 for travel cost: $0, $2 and $5, which approximate the cost of public transportation as well as the effect of a potential travel subsidy that offsets travel costs. A key component of the program was the inclusion of financial incentives. As is common in programs of this sort, various types of financial incentives were offered (cash, supermarket vouchers, Medisave (health savings account) credits and sporting goods vouchers) with the amount of incentive ranging from $150 to $450 over the 6-month period. Finally, a one-time enrolment fee which varied from $0 (a free program), $20 and $50 was also included to quantify the effect of these fees on program participation.

**Figure 1:**
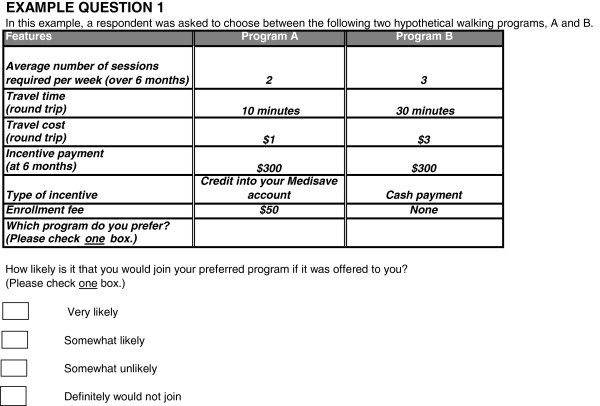
Sample conjoint task.

For each of the ten pairs of hypothetical walking programs, participants were asked to choose their preferred program and then indicate how likely they would be to join the program if it were offered to them. The response options consisted of four choices: “Very likely”, “Somewhat likely”, “Somewhat unlikely” and “Very unlikely”. An example choice task is reproduced in Figure 1. The full survey is available upon request.

### Experimental design

Since our survey contained 6 attributes (each with either 3 or 4 levels), it was not feasible to show respondents every possible combination of attributes and levels. Therefore, following common practice in DCEs [26], we produced a statistically efficient fractional design that was orthogonal, had minimal level overlap and ensured level balance (each level appears approximately the same number of times) [27]. The design consisted of four choice sets with ten tasks each that were allocated randomly to respondents. Each task consisted of two hypothetical walking programs. The survey also included a “rationality” test to determine whether respondents chose the correct alternative in a dominated task (where one choice is the same or better on all attribute levels). Those who chose the dominated alternative were excluded from the analysis (19.8% of the sample) out of concerns that their data may be invalid. This left a sample of 802 respondents.

### Ethic statement

The study was approved by the National University of Singapore IRB, and was performed in accordance with the Declaration of Helsinki.

### Statistical analysis

A random utility model (RUM) was used to analyze responses to the conjoint survey tasks. This model assumes that the utility associated with each program is a function of its attribute levels:

$$u_{\mathrm{jt}}^{i}=v^{i}\left(x_{\mathrm{jt}},c_{\mathrm{jt}}\right)+e_{\mathrm{jt}}^{i}$$

In this specification, $u_{\mathrm{jt}}^{i}$ represents a respondent *i’s* utility for scenario *j* in *t* tasks. *v*^*i*^ represents the observable components of the utility function and is composed of a vector of all program attribute levels excluding the incentive payment (*x*_*jt*_) and the incentive payment (*Cjt*). A model which specified incentive payment levels as categorical (indicator variables for $150, $200, $300 and $450) was found to produce estimates that were not significantly different from those generated from a linear specification. For ease of exposition, the incentive payment was coded as a linear term. All other attributes were effects-coded. Effects coding allows for easy comparisons of the relative importance of the levels as the sum of the preference weights within an attribute will be zero. Linear coding yields a single coefficient for an attribute. In this case, the coefficient on the incentive payment term is assumed to be linear within the range of incentives offered, thus allowing the coefficient to be interpreted as the marginal utility of an additional dollar of incentives. $e_{\mathrm{jt}}^{i}$ is a random error term representing the component of utility which is unobservable. As per utility theory, each respondent is expected to choose the scenario that provides the highest level of utility in each task. This model was estimated using a hierarchical Bayes estimation procedure [28] (implemented in Sawtooth Software CBC Hierarchical Bayes Module version 5.2.8.), which assumes heterogeneous preferences and allows for the estimation of the mean and variance of individual-level preference weights (the Beta coefficients). To derive the monetary amount required for an individual to be indifferent between two attribute levels, we divided the difference in preference weights between the two levels by the marginal utility of the incentive payment.

The inclusion of a follow-up question indicating likelihood of joining a program enabled us to estimate program uptake. To estimate uptake for select programs, we quantified the percentage of individuals whose predicted utility for a given program was greater than their predicted utility when not joining (responding Very Unlikely or Somewhat Unlikely to join). If greater, they were assumed to join the program. We present results for a free program and for one with a $50 enrolment fee. In addition to the above, we also present the proportion of those enrolling who were already meeting activity guidelines, as incentives for these individuals merely represents a transfer payment and are not expected to increase activity levels.

## Results

Table 2 shows the demographic characteristics of our sample. The median age was 59.5 years. Over half the respondents were female and had less than primary school education. Consistent with the population in Singapore, the vast majority were Chinese. Slightly under half were employed yet nearly 80% did not meet activity guidelines.

**Table 2:** Descriptive statistics

***Socio-demographics***	**Age (median)**	**59.5 years**
	Male	45.5%
	Female	54.5%
	Primary school or less	55.5%
	Secondary school or more	44.5%
***Ethnicity***	Chinese	79.1%
	Indian	11.5%
	Malay	8.7%
	Other	0.7%
***Employment status***	Employed	48.6%
	Unemployed	28.7%
	Not in labor force	22.7%
***Physical activity levels***	Meeting guidelines - “Active”	20.6%
	Not meeting guidelines - “Inactive”	79.4%
***Monthly household income***	Under $4,000	50.7%
	$4,000 and above	31.6%
	Not reported	17.7%

**Figure 2:**
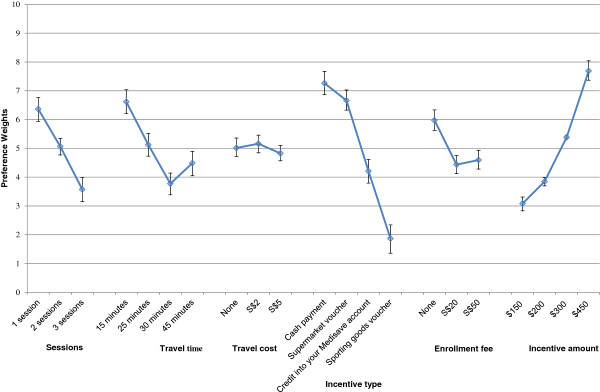
Preferences across attributes and levels.

Regression results from the conjoint analysis are presented in Additional file 1: Table S1. Figure 2 graphically presents these results after centering the preference weights to 5 and rescaling to between 0 and 10 for ease of exposition. Attribute levels with higher preference weights were preferred to those with lower weights. The most preferred walking program was a convenient, free (in terms of travel cost and enrolment fee) program with one 45-minute session per week and a $450 cash incentive. When statistically significant, the attribute levels were in the expected direction. None of the levels for travel cost were significantly different from one another nor was there a difference between the $20 and $50 enrolment fee. In terms of incentive type, cash payments were the most preferred incentive but not significantly different from NTUC supermarket vouchers. Both were preferred to Medisave (health savings) credits and sporting goods vouchers were least preferred.

The relative value of various types of incentives can be seen in Table 3. One dollar in cash is roughly equivalent to $1.09 (95% CI: $1.01 to $1.18) in supermarket vouchers, $1.73 ($1.52 to $1.98) in Medisave vouchers, and $3.89 ($2.99 to $5.54) in sporting good vouchers. All of the differences are statistically significant.

**Table 3:** Incentive amount needed by payment type

**Payment type**	**Incentive amount equivalent to $1 in cash (95% CI)**
Cash payment	$1.00 (-)
Supermarket voucher	$1.09 ($1.01 - $1.18)
Credit into your medisave account	$1.73 ($1.52 - $1.98)
Sporting goods voucher	$3.89 ($2.99 - $5.54)

**Figure 3:**
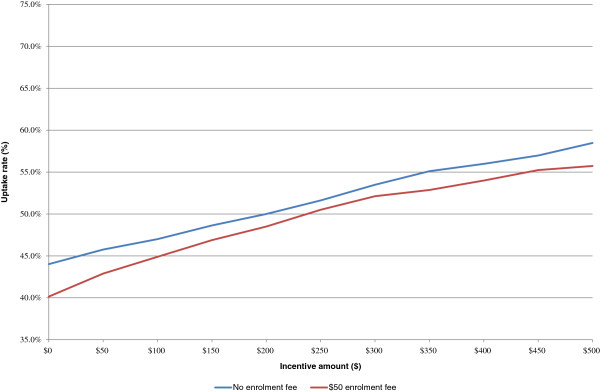
Uptake rate by enrolment fee for varying incentive amounts.

Figure 3 shows estimated uptake for the sample program shown in Table 4 with 1) no enrolment fee and 2) a $50 enrolment fee. As expected, the free program results in higher predicted uptake at all incentive levels, and greater incentives generate greater stated levels of enrolment. However, including a $50 enrolment fee only decreases enrolment by an average of 2%. Based on these results, a free program with $500 worth of incentives would generate enrolment rates of 58.5%; charging $50 to enroll only lowers this figure to 55.7%.

**Table 4:** Hypothetical walking program used to estimate uptakes

**Attribute**	**Level**
Sessions	3 sessions
Travel time	25 minutes
Travel cost	S$2
Incentive payment	*Varies*
Incentive type	Cash payment
Enrollment fee	*Varies*

**Figure 4:**
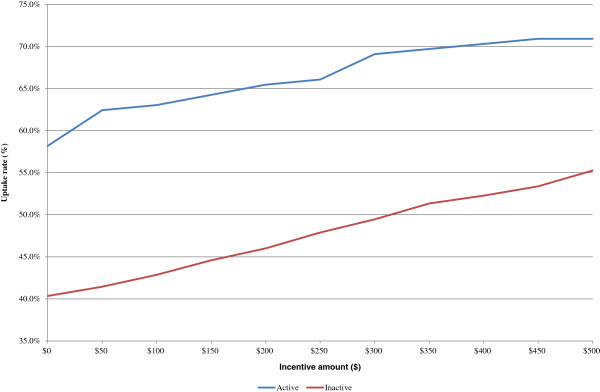
Uptake rate by physical activity status for varying incentive amounts.

Figure 4 shows how uptake varies between those who state they are meeting physical activity guidelines (”Active”) and those who state they are not (“Inactive”). Almost half of Actives claim they would participate in the sample program even if they were not offered any incentives. The percentage of actives increases to as much as 69% for the highest incentive amount. In contrast, only 38% of Inactives claim they would join a free program. However, when incentives are provided, their stated participation increases at a faster rate than for Actives. At $500, over 55% of Inactives state they would join the program. Because they are the majority of the population, regardless of the incentive amount, Inactives represent at least 72% of participants, and this number increases with higher incentive levels.

## Discussion

The objective of this study was to identify a promising walking program for older, multi-ethnic adults in Singapore, and then to test the effects on stated enrolment when incentives and/or an enrolment fee are offered. Not surprisingly, results showed that respondents most prefer the least onerous program with the greatest benefits. This is one involving minimal travel time and cost, comprising the least number of weekly activity sessions (1 session per week), offering a 450SGD cash incentive and without any enrolment fee. Although minimal, one brisk walking session per week would account for 30% of recommended weekly activity if participants walk at the recommend pace. Moreover, this level of activity is still associated with some health benefits, especially among sedentary adults [29-31]. Therefore, it may be worth offering such a program, even with incentives, if it could encourage sustained increases in activity levels.

A key finding of this study is that financial incentives increase predicted uptake for all programs offered and more so among the least active older adults. For the hypothetical six month program offered, providing SGD$150 for meeting participation goals (SGD$25 ≈ USD20 monthly over 6 months assuming 1SGD = 1.25USD) increases predicted program uptake by 8.5% (assuming no enrolment fee). Raising this to SGD$450 (SGD$75 ≈ USD 60 monthly over 6 months) increases uptake by 13%. These results demonstrate the potential for even modest incentives to increase uptake. Moreover, although cash was clearly the most preferred option, supermarket vouchers, which could potentially be purchased in bulk at a discount, were a close alternative. Health savings account credits and sporting goods vouchers/equipment are not recommended as respondents place a lower value on these rewards and thus they are less of a motivator.

Although we show that incentives increase program uptake and have been shown to be successful in improving outcomes in prior activity studies [25,32], they are not without cost. One way to offset those costs is via an enrolment fee. These results suggest that an enrolment fee is a viable option as it has a minimal impact on participation. Moreover, the largest impact occurs when going from a free program to a program with a nominal fee. Increases beyond this level can be set so as to recover the cost of the incentives, and will likely have only a modest additional loss in participation; less than 2% based on our estimates when fees are within the $SGD20 to $SGD50 range. Moreover, if the enrolment fee is seen as a deposit whose return is dependent upon program completion, it is likely to have the added benefit of reducing attrition and increasing program effectiveness [21].

When incentives are provided, there is always the concern of adverse selection. In this case the concern is that the incentives go to those who would have been doing the required amounts of activity even without the incentives. Although it is unclear whether these results would generalize to other older adult populations, we found that incentives have a *greater* stated effect on enrolment for inactive participants. This result may not be surprising given that active participants may have their routines well established and may be less interested in these types of programs. A financial incentive may also be less of a motivation to them, as they have already made the decision to be active. Nevertheless, active participants may benefit through accruing more health benefits to the extent that incentives induce additional physical activity [33-35]. Monetary incentives are likely to have the greatest effect on participation and outcomes for those who are least active.

Although the first of its kind in a multi-ethnic sample of older adults, this study adds to growing evidence of the potential for financial incentives to motivate health behavior change [24,25,36,37], specifically in promoting activity among older adults in the US and other countries. These results are corroborated by Finkelstein et al. [25] who observed significant, large effects in an incentive-based physical activity trial among older adults aged 50+ where participants assigned to the intervention arm logged an average of 4.1 hours of activity a week relative to their counterparts in the control arm who logged only 2.3 hours over 4 weeks. However, several limitations must be noted. The primary limitation is that the results are based on a survey, and not a real world experiment. The extent to which these surveys serve as a good basis for successful program implementation remains unknown. This should be an area of future research. As such, the results should be interpreted with caution. Yet this is both a limitation and a strength. This study used a low cost survey elicitation technique to generate hypotheses about what might happen were these programs to be offered; future studies should test these predictions in the real world. Moreover, as with any study, even if accurate for Singapore, the generalizability of these results beyond older adults here is unknown. Future studies will need to be targeted to the population of interest. Besides, field studies involving older adults would also need to account for the unique cognitive and psychosocial characteristics of this sub-population [38]. Also, real world cost-effectiveness studies would be a necessary next step to evaluate the long-term feasibility of such programs in specific settings. Finally, incentives, even if effective at increasing uptake, may not be sustainable as they may not translate into sustained behavior change. However, in at least one study, increased activity persisted even after the removal of incentives [39,40].

The use of incentives has raised concerns about a “crowding out effect”. This would result if the incentives undermine intrinsic motivation. Yet, although theory suggests this could be the case, there is no empirical evidence to support it [41]. Several studies show that incentives increase activity levels of both children and adults. As a result, this appears not to be a legitimate concern [25,32,40]. Also, providing incentives may not be affordable in the long term. Although we show that an enrolment fee is one strategy to overcome this concern, other strategies will be required to engage sedentary individuals for whom incentives are ineffective. Identifying these strategies should be a topic of future research. Regardless, these results suggest that incentives may be part of a comprehensive solution aimed at increasing activity levels of older adults.

## Conclusions

Study findings add to the growing literature that incentives are a feasible and potentially effective strategy to increase program uptake among older adults. Although cash is the most preferred incentive type, supermarket vouchers are a close alternative. To offset associated costs, enrolment fees are a viable option. Future research should assess the affordability of incentive use and sustainability of model programs in addition to identifying other strategies to encourage sustained activity of older adults.

## Abbreviations

ACSM: America college of sports medicine; AHF: American heart foundation; NCD: Non-communicable diseases; HPB: Health promotion board; RUM: Random utility model.

## Competing interests

The authors declare that they have no competing interests.

## Authors’ contributions

EAF, MAF and YTT contributed to the conception and design of the project. MAF performed all analyses and MB assisted with interpretation. MAF and YTT completed the first draft while EAF provided critical revision. All authors edited and approved the final manuscript.

## Pre-publication history

The pre-publication history for this paper can be accessed here:

http://www.biomedcentral.com/1471-2458/14/141/prepub

## Supplementary Material

Additional file 1: Table S1 Regression results. Click here for file
