# Supplementary material for: Effects of financial incentives on motivating physical activity among older adults: results from a discrete choice experiment
Source: BMC Public Health. 2014 Feb 10;14:141. doi: 10.1186/1471-2458-14-141 (PMC3933254; doi:10.1186/1471-2458-14-141)
Supplement: Additional file 1: Table S1 — Regression results. [file 1471-2458-14-141-S1.pdf]

Table S1. Regression results

Attribute	Levels	Hierarchical Bayes coefficient estimate (95% Confidence Interval)
Number of sessions	1 session	0.54 (0.37 to 0.71)
	2 sessions	0.03 (-0.09 to 0.14)
	3 sessions	-0.57 (-0.74 to -0.4)
Travel time	15 minutes	0.65 (0.48 to 0.81)
	25 minutes	0.05 (-0.11 to 0.21)
	30 minutes	-0.49 (-0.64 to -0.34)
	45 minutes	-0.21 (-0.38 to -0.04)
Travel cost	None	0.01 (-0.11 to 0.14)
	S$2	0.06 (-0.06 to 0.18)
	S$5	-0.07 (-0.17 to 0.04)
Incentive type	Cash payment	0.91 (0.75 to 1.07)
	Supermarket voucher	0.66 (0.53 to 0.81)
	Credit into your Medisave account	-0.32 (-0.48 to -0.16)
	Sporting goods voucher	-1.25 (-1.46 to -1.06)
Enrollment fee	None	0.39 (0.25 to 0.53)
	S$20	-0.23 (-0.35 to -0.1)
	S$50	-0.16 (-0.29 to -0.03)
Incentive payment	(Linear)	6.14 (5.41 to 6.95)
Not joining	(Linear)	2.24 (1.68 to 2.81)
